# Computer-Based Tools Unmask Critical Mineral Nutrient Interactions in Hoagland Solution for Healthy Kiwiberry Plant Acclimatization

**DOI:** 10.3389/fpls.2021.723992

**Published:** 2021-10-28

**Authors:** Sara Maleki, Bahram Maleki Zanjani, Bahram Baghban Kohnehrouz, Mariana Landin, Pedro Pablo Gallego

**Affiliations:** ^1^Department of Agronomy and Plant Breeding, Faculty of Agriculture, University of Zanjan, Zanjan, Iran; ^2^Agrobiotech for Health, Department of Plant Biology and Soil Science, Faculty of Biology, University of Vigo, Vigo, Spain; ^3^Department of Plant Breeding and Biotechnology, University of Tabriz, Tabriz, Iran; ^4^Department of Pharmacology, Pharmacy and Pharmaceutical Technology Department, Grupo I+D Farma (GI-1645), Faculty of Pharmacy, University of Santiago, Santiago de Compostela, Spain

**Keywords:** *Actinidia arguta*, artificial intelligence, *ex vitro* acclimatization, DOE, kiwiberry, machine learning, healthy plants, physiological disorders

## Abstract

The aim of this study was to better understand the response of *ex vitro* acclimatized plants grown to a set of mineral nutrient combinations based on Hoagland solution. To reach that, two computer-based tools were used: the design of experiments (DOE) and a hybrid artificial intelligence technology that combines artificial neural networks with fuzzy logic. DOE was employed to create a five-dimensional IV-design space by categorizing all macroelements and one microelement (copper) of Hoagland mineral solution, reducing the experimental design space from 243 (3^5^) to 19 treatments. Typical growth parameters included hardening efficiency (Hard), newly formed shoot length (SL), total leaf number (TLN), leaf chlorophyll content (LCC), and leaf area (LA). Moreover, three physiological disorders, namely, leaf necrosis (LN), leaf spot (LS), and curled leaf (CL), were evaluated for each treatment (mineral formulation). All the growth parameters plus LN were successfully modeled using neuro-fuzzy logic with a high train set *R*^2^ between experimental and predicted values (72.67 < *R*^2^ < 98.79). The model deciphered new insights using different sets of “IF–THEN” rules, pinpointing the positive role of Mg^2+^ and Ca^2+^ to improve Hard, SL, TLN, and LA and alleviate LN but with opposite influences on LCC. On the contrary, TLN and LCC were negatively affected by the addition of NO_3_^–^ into the media, while NH_4_^+^ in complex interaction with Cu^2+^ or Mg^2+^ positively enhanced SL, TLN, LCC, and LA. In our opinion, the approach and results achieved in this work are extremely fruitful to understand the effect of Hoagland mineral nutrients on the healthy growth of *ex vitro* acclimatized plants, through identifying key factors, which favor growth and limit physiological abnormalities.

## Introduction

The first studies on the development of mineral nutrient solutions for the cultivation of healthy plants date back to the mid-1800s. They are mainly based on physiologically balanced formulations, i.e., Knop formulation (Loew and Aso, 1907). This approach was substituted by testing serial concentrations of elements of Knop. In this case, a similar osmotic concentration for all the media is tested, but each formulation differs from the others in the proportions of nutrient salts (Tottingham, 1914; Shive, 1915; Jones and Shive, 1921). Subsequently, the variation in the pH range of the formulated mineral solution was introduced (Arnon and Johnson, 1942).

Hoagland and colleagues soon reported outstanding improvements in mineral formulations for healthy commercial plant growth (Hoagland, 1920, 1937; Hoagland and Broyer, 1936; Arnon, 1937; Arnon and Hoagland, 1939), but also some associated physiological problems with those formulations (Hoagland and Arnon, 1941, 1948). In 1950, they established the most widely cited and used plant mineral nutrition formulation: Hoagland solution (Hoagland and Arnon, 1950). The new formulation was based on the quantification of the nutrients absorbed by the plant roots followed mainly by the substation of several mineral nutrients until satisfactory results were obtained (Hoagland, 1920; Arnon, 1938). This procedure made the task time-consuming, intensive, and laborious (Hoagland and Arnon, 1950 and references therein).

Although the Hoagland solution has been commonly used for several crops with good results, *ex vitro* culture of plants is still one of the most important challenges for researchers. For each case, different strengths of Hoagland solution need to be applied or its elements must be readjusted. For instance, a different strength of Hoagland solution (0.125-2×) was tested by Kang and Iersel (2004) for growing *Salvia splendens*, pinpointing 1–2 × as optimum levels for healthy plant growth. The half strength of Hoagland solution was determined as the best level for the healthy growth of *Citrus* sp. (Zhou et al., 2020). Also, the serial concentrations of Ca^2+^ and BO_3_^–^ were tested based on the half strength of Hoagland to improve the healthy growth of *Actinidia deliciosa* L. (Sotiropoulos et al., 1999). However, finding an optimized solution of mineral nutrients remains a time-consuming, costly, and tedious task (Nezami-Alanagh et al., 2014).

Recently, computer-based tools for the design of experiments (DOE) made it possible to drastically reduce the number of combinations to be studied compared with traditional factorial designs (Niedz and Evens, 2016). Although DOE has been widely used to improve *in vitro* plant tissue culture (PTC) practice (Niedz and Evens, 2007; Poothong and Reed, 2015; Ayuso et al., 2017; Nezami-Alanagh et al., 2018, 2019; Chu et al., 2019; Hameg et al., 2020; Pence et al., 2020), as far as we know, there is no report regarding the application of the DOE to ensure adequate sampling of the design space in plant mineral solution formulations.

The next challenge of mineral nutrient studies is understanding ion-specific effects in n-dimensional design spaces. In this situation, the use of artificial intelligence (AI) tools has been recommended as a suitable alternative computational methodology to extract information from complex databases (Gago et al., 2010a; Gallego et al., 2011). Neuro-fuzzy logic is one of the powerful multiscale analysis systems of AI technology with the ability to model non-linear complex systems by setting simple “IF–THEN” rules together with the identification of the key factors to improve a specific response (Landin et al., 2009). In recent years, several studies have proved the efficacy of neuro-fuzzy logic in *in vitro* culture media improvements (Nezami-Alanagh et al., 2014, 2017; Ayuso et al., 2017), but despite these advances, the development of an optimal mineral formulation for plant growth continues to be a challenge.

Here, the commercially important kiwiberry or hardy kiwi (Atkinson and Macrae, 2007) plants were selected to establish the response of micropropagated plants to a set of mineral formulations during the *ex vitro* acclimatization (hardening) process. To that end, we implemented DOE to generate a multifactor design space to simultaneously study the influence of Hoagland mineral nutrients on a set of physiological responses, followed by applying neuro-fuzzy logic to model and unveil the key mineral nutrients to decipher hidden relationships between mineral nutrients and the studied parameters.

## Materials and Methods

### Plant Material and *in vitro* Culture Conditions

Micropropagated plants of *Actinidia arguta* (Sieb. and Zucc.) Planch. ex Miq. cv. “Issai” were obtained from the Department of Plant Biology and Soil Sciences (University of Vigo) as described in detail elsewhere (Hameg, 2019; Hameg et al., 2020). Briefly, micro-shoots were proliferated in Cheng medium (Cheng, 1975) containing 1 mg/L N6-benzyladenine (BAP), 1 mg/L gibberellic acid (GA3), 30 g/L sucrose, and 8 g/L agar. Medium pH was adjusted to 5.7 prior to autoclaving (121°C, 1 kg/cm^2^/s for 20 min). The cultures were kept under 16-h photoperiod (white fluorescent tubes; irradiance of 40 μmol/m^2^/s) and day/night temperature of 25 ± 1°C and cultured for 50 days (Hameg et al., 2018).

### Direct *ex vitro* Simultaneous Rooting and Acclimatization Culture Conditions

Micro-shoots (∼3 cm in height) obtained from *in vitro* proliferation medium, after dipping the basal cut end of the micro-shoots in 250 ppm indole-3-butyric acid (IBA) solution for 10 min, were carefully planted into mini-pots (5 × 5 cm^2^) containing perlite, covered with glass vessels. The mini-pots were transferred into an automated growth room (Sanyo SGC066.CFX.F) under 16-h photoperiod (white fluorescent tubes; irradiance of 200 μmol/m^2^/s) and 18 ± 3°C. The initial value of relative humidity was set at 100% and decreased gradually during 21 days until 60% was reached (Gago et al., 2010b).

Thereafter, rooted plantlets were watered for 3 months with a set of mineral formulations (Table 1), based on the Hoagland solution (Hoagland and Arnon, 1950). Each replicate consisted of two transplant plastic containers each containing 10 plants. The experiments were carried out in duplicate.

**TABLE 1:** Composition of the mineral formulations established by the five-factor design (19 treatments) based on Hoagland mineral nutrients plus half strength of Hoagland solution (as control) in mg/L.

Formulations	NH_4_H_2_PO_4_	Ca(NO_3_)_2_.4H_2_O	KNO_3_	MgSO_4_.7H_2_O	CuSO_4_.5H_2_O
A	57.515	944.6	6.066	492.9	0.0008
B	115.03	472.3	303.3	246.45	0.08
C	1.1503	9.446	606.6	4.929	0.04
D	1.1503	944.6	303.3	246.45	0.04
E	57.515	9.446	6.066	4.929	0.08
F	115.03	472.3	606.6	492.9	0.0008
G	57.515	944.6	303.3	492.9	0.0008
H	1.1503	9.446	606.6	246.45	0.08
I	115.03	472.3	6.066	4.929	0.04
J	1.1503	9.446	606.6	492.9	0.08
K	115.03	944.6	6.066	246.45	0.0008
L	57.515	472.3	303.3	4.929	0.04
M	57.515	944.6	6.066	492.9	0.08
N	1.1503	472.3	606.6	246.45	0.04
O	1.1503	472.3	303.3	492.9	0.08
P	115.03	944.6	606.6	246.45	0.04
Q	115.03	472.3	6.066	246.45	0.0008
R	1.1503	9.446	606.6	492.9	0.04
S	57.515	944.6	303.3	4.929	0.08
Control	57.515	472.3	303.3	246.45	0.04

*Three levels were selected for each factor (0.01, 0.5, and 1 × Hoagland solution).*

### Experimental Design and Data Acquisition

An experimental design was established to study the effects of the combination of six macronutrients (i.e., N, P, Ca, K, S, and Mg) and one micronutrient (Cu) of the Hoagland solution (Hoagland and Arnon, 1950) on the growth and development of acclimatized plants. To this end, the Hoagland salts, namely, (i) NH_4_H_2_PO_4_, (ii) KNO_3_, (iii) Ca(NO_3_)_2_.4H_2_O, (iv) MgSO_4_.7H_2_O, and (v) CuSO_4_.5H_2_O, were considered as five independent factors at three levels, expressed as × half-strength Hoagland solution concentrations (Table 1) for ensuring optimal sampling of the design space. In other words, here we select the space delimited by three levels of Hoagland solution varied between 0.01, 0.5, and 1 (×level). This means that various formulations of Hoagland solution were tested: some at a very low concentration of all ions (0.01×); others at middle concentrations (0.5 × level) similar to the control (½ strength), and the rest at full-strength Hoagland solution (1 × level).

The five-factor experimental design was a 19-point using IV-optimal response surface and the software application Design-Expert^®^8 (Design-Expert, 2010), and another point with half-strength Hoagland salt concentration as control (Table 1). The other salts in the Hoagland solution, including micronutrients and iron, were fixed based on the control medium (Supplementary Table 1).

Samples of plants were irrigated with each mineral formulation. After 3 months, five growth responses and three physiological disorders were evaluated:

1. Hardening efficiency (Hard): percentage of successfully acclimatized plants.
2. Shoot length (SL): length of shoots in cm.
3. Total leaf number (TLN): number of leaves.
4. Leaf chlorophyll content (LCC): measured by SPAD chlorophyll meter (Opti-Sciences CCM-200, United States), expressed as Chlorophyll Content Index (CCI).
5. Leaf area (LA): measured by CI-202 Laser Leaf Area Meter in cm^2^.
6. Leaf necrosis (LN): number of necrotic leaves per total leaf numbers in %.
7. Curled leaf (CL): number of curled leaves per total leaf numbers in %.
8. Leaf spot (LS): number of spotted leaves per total leaf numbers in %.

### Artificial Neural Networks Modeling Tool

FormRules^®^ v4.03 (Intelligensys Ltd., United Kingdom), a neuro-fuzzy logic software that combines artificial neural networks with fuzzy logic, was used to model the results and analyze how the solution components modulate its physiological effects through the simple “IF–THEN” rules that generate with a membership degree, as described in detail previously (Colbourn and Rowe, 2005; Gallego et al., 2011; Landin and Rowe, 2013; Nezami-Alanagh et al., 2018).

Different statistical criteria of software fitting, namely, Cross validation (CV), Leave One Out Cross Validation (LOOCV), Minimum Description Length (MDL), Bayesian Information Criterion (BIC), and the Structural Risk Minimization (SRM), were tested to build the model. Among them, SRM was selected, because it generated the most predictable models along with the minimum generalization error and the simplest and more intelligible rule sets avoiding overtraining (Vapnik, 1992; Shao et al., 2006). All data were used for training since SRM is a statistical significance method; the number of subsets ranged from 1 to 3, and a maximum of 4 inputs per submodel and 15 maximum nodes per input were selected (Table 2) as described elsewhere (García-Pérez et al., 2020; Hameg et al., 2020).

**TABLE 2:** Training parameters setting with neuro-fuzzy logic.

Critical factors for neuro-fuzzy logic model
*Minimization parameters*
Ridge regression factor: 1e^–6^
*Model selection criteria*
Structural risk minimization (SRM)
C1 = 0.75–0.95; C2 = 4.8
Number of set densities: 2
Set densities: 2, 3
Adapt *nodes*: True
Max. *inputs* per submodel: 4
Max. *nodes* per *input*: 15

The model quality was assessed using the coefficient of determination of the training set (train set *R*^2^), expressed in percentage (for model predictability), and the analysis of variance (ANOVA) parameters (for model accuracy). Train set *R*^2^ values are calculated by the following equation (Shao et al., 2006):


$$R^{2}=\left(1-\frac{\sum_{i=1}^{n}{\left(y_{i}-y_{i}^{\prime }\right)}^{2}}{\sum_{i=1}^{n}{\left(y_{i}-y_{i}^{"}\right)}^{2}}\right)\times 100\%$$

where *y*_*i*_ is the experimental value in the database, *y*_*i*_′ is the predicted value generated by the model, and *y*_*i*_″ is the mean of the dependent variable.

Train set *R*^2^ values between 70 and 99.9% are indicative of acceptable predictabilities, while values higher than 99.9% have been rejected due to model over fitting (Colbourn and Rowe, 2005; Landin et al., 2009). To test model accuracy, the software uses one-way ANOVA to evaluate statistical differences between predicted and experimental data. Models, which computed ratio *f*-value in the ANOVA greater than critical *f*-value for the corresponding degrees of freedom (α = 0.01), show good accuracy and no statistical differences among experimental and predicted values (Colbourn and Rowe, 2009).

Modeling was built according to the methodology described previously by Nezami-Alanagh et al. (2017) using the training parameters shown in Table 2.

To avoid the ion confounding effect (Niedz and Evens, 2007), the mineral composition of every formulation was expressed as ion concentrations instead of salt concentrations and introduced as *inputs* (NH_4_^+^, Ca^2+^, K^+^, Mg^2+^, NO_3_^–^, PO_4_^2–^, SO_4_^2–^, and Cu^2+^), while physiological parameters (Hard, SL, TLN, LCC, LA, LN, LS, and CL) were selected as *outputs*.

## Results

The reduced experimental design allowed establishing just 19 treatments (mineral formulations) using different proportions of the mineral nutrients of Hoagland solution in a well-sampled design space. As it can be observed (Table 3), while some formulations promoted the highest plant survival (100% Hard) such as A and B, others such as C, F, H, J, and R were completely unviable (0% Hard), all plants showing 100% of leaf necrosis (C, H, J, and R) or 100% of spots (F) and dying during 3 months of acclimatization. Moreover, some other formulations (e.g., B, G, I, L, P, and S) and the control (½ Hoagland strength) also promoted rates of physiological disorders such as leaf spots, curling, and/or necrosis (Table 3). Thus, it is clear that the mineral composition of each formulation assayed plays an essential role in plant survival during the acclimatization procedure. However, these data are not very informative about which component of each formulation caused those positive or negative effects on plant growth and health, thus not much valuable knowledge can be drawn from these results.

**TABLE 3:** Dataset used to build the neuro-fuzzy model, including the mineral ion composition as inputs and the experimental data obtained for each growth response and physiological disorder parameters (expressed as the mean ± standard error) as outputs.

																
Formulations	Ions (mM)								Physiological disorders							
	
	NH_4_^+^	NO_3_^–^	K^+^	Ca^2+^	Mg^2+^	Cu^2+^	PO_4_^2–^	SO_4_^2–^	Hard (%)	SL (cm)	TLN	LCC	LA (cm^2^)	LN (%)	CL (%)	LS (%)
																
A	0.50	8.06	0.06	4.00	2.00	0.0000	0.50	2.05	100	6.28 ± 0.50	31.45 ± 2.036	22.39 ± 1.21	11.14 ± 1.70	0.00	2.54 ± 0.98	0.65 ± 0.38
B	1.00	7.00	3.00	2.00	1.00	0.0003	1.00	1.05	100	7.35 ± 0.26	56.80 ± 2.40	34.45 ± 4.85	8.98 ± 0.75	13.62 ± 3.69	6.99 ± 1.10	0.00
C	0.01	6.08	6.00	0.04	0.02	0.0002	0.01	0.07	0.00	N/D	N/D	N/D	N/D	100	N/D	N/D
D	0.01	11.00	3.00	4.00	1.00	0.0002	0.01	1.05	60.00 ± 24.50	2.13 ± 0.07	12.33 ± 2.60	3.57 ± 0.41	0.98 ± 0.15	0.00	0.00	100
E	0.50	0.14	0.06	0.04	0.02	0.0003	0.50	0.07	25.00 ± 9.00	2.40 ± 0.23	11.33 ± 1.20	14.63 ± 1.20	3.15 ± 0.25	0.00	0.00	36.27 ± 2.56
F	1.00	10.00	6.00	2.00	2.00	0.0000	1.00	2.05	0.00	N/D	N/D	N/D	N/D	N/D	N/D	100
G	0.50	11.00	3.00	4.00	2.00	0.0000	0.50	2.05	40.00 ± 16.33	3.83 ± 0.48	46.50 ± 4.92	14.80 ± 1.05	4.41 ± 0.88	0.00	6.50 ± 2.28	0.00
H	0.01	6.08	6.00	0.04	1.00	0.0003	0.01	1.05	0.00	N/D	N/D	N/D	N/D	100	N/D	N/D
I	1.00	4.06	0.06	2.00	0.02	0.0002	1.00	0.07	83.33 ± 11.24	2.74 ± 0.38	19.40 ± 1.59	10.62 ± 1.24	1.79 ± 0.37	0.00	0.00	68.57 ± 6.80
J	0.01	6.08	6.00	0.04	2.00	0.0003	0.01	2.05	0.00	N/D	N/D	N/D	N/D	100	N/D	N/D
K	1.00	8.06	0.06	4.00	1.00	0.0000	1.00	1.05	92.31 ± 7.69	2.97 ± 0.41	23.58 ± 2.92	20.91 ± 0.69	3.83 ± 1.93	0.00	0.00	0.00
L	0.50	7.00	3.00	2.00	0.02	0.0002	0.50	0.07	66.67 ± 21.08	1.38 ± 0.43	19.75 ± 1.60	9.23 ± 0.99	2.24 ± 0.67	0.00	0.00	80.75 ± 7.26
M	0.50	8.06	0.06	4.00	2.00	0.0003	0.50	2.05	75.00 ± 9.93	6.25 ± 0.82	33.87 ± 2.52	16.17 ± 1.35	8.81 ± 1.19	0.00	0.00	20.45 ± 5.96
N	0.01	10.00	6.00	2.00	1.00	0.0002	0.01	1.05	87.50 ± 12.5	1.80 ± 0.31	17.43 ± 1.63	7.04 ± 0.30	1.01 ± 0.12	0.00	0.00	25.74 ± 15.65
O	0.01	7.00	3.00	2.00	2.00	0.0003	0.01	2.05	71.43 ± 18.44	2.64 ± 0.69	11.80 ± 2.35	6.76 ± 0.65	1.20 ± 0.25	0.00	0.00	62.99 ± 9.73
P	1.00	14.00	6.00	4.00	1.00	0.0002	1.00	1.05	42.86 ± 20.20	3.65 ± 0.49	34.00 ± 1.15	11.70 ± 3.40	5.74 ± 2.87	16.04 ± 2.00	0.00	0.00
Q	1.00	4.06	0.06	2.00	1.00	0.0000	1.00	1.05	83.33 ± 16.67	3.90 ± 0.42	17.60 ± 1.17	19.98 ± 2.13	9.20 ± 3.01	0.00	0.00	0.00
R	0.01	6.08	6.00	0.04	2.00	0.0002	0.01	2.05	0.00	N/D	N/D	N/D	N/D	100	N/D	N/D
S	0.50	11.00	3.00	4.00	0.02	0.0003	0.50	0.07	87.50 ± 12.5	1.75 ± 0.19	17.43 ± 0.97	6.63 ± 0.23	1.48 ± 0.14	0.00	0.00	71.81 ± 6.34
Control	0.50	7.00	3.00	2.00	1.00	0.0002	0.50	1.05	71.11 ± 6.83	5.19 ± 0.26	44.44 ± 1.80	15.33 ± 0.84	6.12 ± 0.67	1.90 ± 0.67	4.37 ± 1.00	0.00

*N/D, not determined.*

**FIGURE 1:**
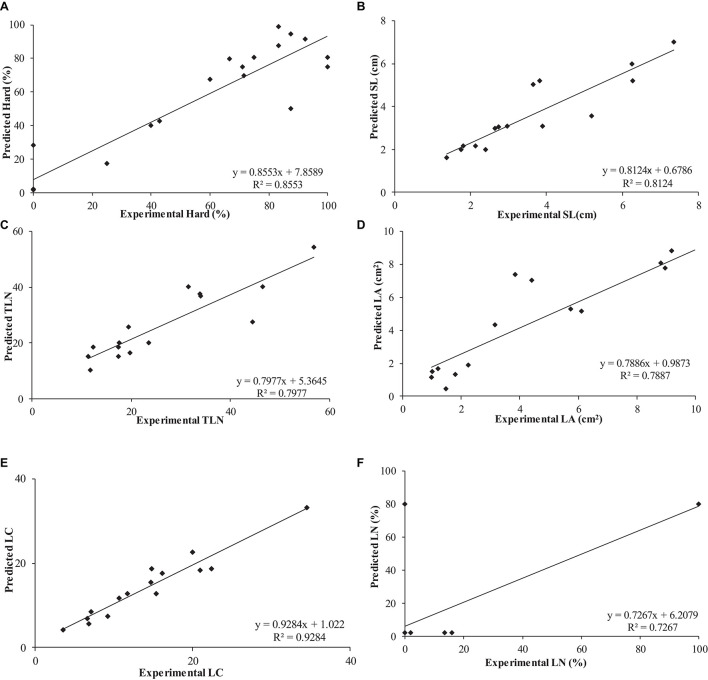
Determination of coefficient (*R*^2^) of experimental vs. predicted values obtained by neuro-fuzzy logic models for the different parameters or *outputs* studied: **(A)** Hard, **(B)** SL, **(C)** TLN, **(D)** LA, **(E)** LCC, and **(F)** LN.

The use of neuro-fuzzy logic models permitted the successful modeling of six out of eight parameters (*outputs*): Hard, SL, TLN, LCC, LA, and LN with high predictability (72.67 ≤ *R*^2^ ≤ 92.84%) and accuracy (*f* ratio > *f* critical). However, the reduced number of degrees of freedom did not allow confirmation of the accuracy of CL and LS models, despite their excellent predictability *R*^2^ > 93.71% (Figure 1 and Table 4).

**TABLE 4:** Neuro-fuzzy logic results for each output model.

Outputs	Submodel	Significant inputs	*R* ^2^	*f* ratio	df1, df2	*f* critical
Hard (%)	**1**	**Mg^2+^× NO_3_** ^–^	85.52	12.80	6, 19	2.91
	2	Ca^2+^				
SL (cm)	**1**	**NH_4_^+^ × Cu^2+^**	81.24	7.79	5, 14	3.84
	2	Mg^2+^				
TLN	**1**	**NH_4_^+^ × Cu^2+^**	79.77	7.10	5, 14	3.84
	2	Mg^2+^				
LCC	**1**	**NH_4_^+^ × Cu^2+^**	92.84	9.73	8, 14	4.15
	2	Mg^2+^				
	3	Ca^2+^				
LA (cm^2^)	1	**NH_4_^+^ × Mg^2+^**	78.87	6.72	5, 14	3.84
	2	NO_3_^–^				
LN (%)	**1**	**Ca^2+^**	72.67	13.29	3, 18	3.29
CL (%)	-	-	93.72	4.07	11, 14	8.76
LS (%)	-	-	98.79	5.81	14, 15	245.28

*The number of submodels, significant inputs, predictability, and accuracy parameters: Train set *R*^2^ and ANOVA parameters for training [*f* ratio, degree of freedom (df1: model and df2: total) and *f* critical value for α = 0.05]. The *input*s with the strongest effect on each *output* are in bold.*

Neuro-fuzzy logic generated 1–3 submodels for each parameter. Two submodels explain the variability of plant survival (Hard): the interaction of NO_3_^–^ and Mg^2+^, highlighted as the *inputs* with the strongest effect, and the independent effect of Ca^2+^. The variance found for SL (81.24) TLN (79.77), and LCC (92.84) was mainly due to the interaction of NH_4_^+^ and Cu^2+^ (stronger effect) and the independent influence of Mg^2+^. For LCC, the independent influence of Ca^2+^ also played an essential role. For LA, the strongest effect was caused by the interaction of NH_4_^+^ and Mg^2+^ and the independent influence of NO_3_^–^. Finally, LN variations are explained by variations in Ca^2+^ ion concentrations (Table 4).

Neuro-fuzzy logic provides a set of useful “IF–THEN” rules to explain those effects and facilitate their understanding using words (linguistic tags), as well as to help researchers in decision-making. Table 5 summarizes all the rules with their membership degree. The rules showing the ion combination with the strongest effect and highest membership (1.00) for each parameter are in bold.

**TABLE 5:** Rules generated by neuro-fuzzy logic showing the best combination of *inputs* to obtain the highest and lowest physiological responses with their membership degree for each *output.*

Rules		NH_4_^+^	NO_3_^–^	Ca^2+^	Mg^2+^	Cu^2+^		Hard	SL	TLN	LCC	LA	LN	Membership
1	IF			Low			THEN	Low						1.00
2				Mid				High						1.00
3				High				High						1.00
4			Low		Low			High						0.96
**5**			**Low**		**High**			**High**						**1.00**
6			High		Low			Low						0.79
**7**			**High**		**High**			**Low**						**1.00**
8	IF				Low		THEN		Low					1.00
9					High				High					1.00
10		Low				Low			Low					0.85
**11**		**Low**				**High**			**Low**					**1.00**
12		High				Low			Low					0.92
**13**		**High**				**High**			**High**					**1.00**
14	IF	Low				Low	THEN			High				0.68
**15**		**Low**				**High**				**Low**				**1.00**
16		High				Low				Low				1.00
**17**		**High**				**High**				**High**				**1.00**
18					Low					Low				1.00
19					High					High				0.98
20	IF	Low				Low	THEN				Low			1.00
21		Low				Mid					Low			1.00
**22**		**Low**				**High**					**Low**			**1.00**
23		High				Low					High			0.84
24		High				Mid					Low			0.70
**25**		**High**				**High**					**High**			**1.00**
26					Low						Low			1.00
27					High						High			1.00
28				Low							High			0.92
29				High							Low			0.91
30	IF	Low			Low		THEN					Low		1.00
31		Low			High							Low		1.00
**32**		**High**			**Low**							**Low**		**1.00**
**33**		**High**			**High**							**High**		**1.00**
34			Low									High		1.00
35			High									Low		0.93
**36**	**IF**			**Low**			**THEN**						**High**	**0.80**
**37**				**Mid**									**Low**	**0.99**
38				High									Low	0.98

*The *input*s with the strongest effect on each *output* are in bold.*

The “IF–THEN” rules for Hard indicate that the highest survival of the acclimatized plants was obtained if a low amount of NO_3_^–^ was supplied into the mineral formulation, particularly if combined with high Mg^2+^ content (rules 4–5; Table 5). Also, the model recommends supplementing the formulation with mid to high amounts of Ca^2+^ (rules 2–3; Table 5). The meaning of low, mid, or high concentrations for the different *inputs* can be found elsewhere (García-Pérez et al., 2020; Hameg et al., 2020).

The analysis of “IF–THEN” rules for SL, TLN, and LCC revealed that only Cu^2+^ and NH_4_^+^ supplied at high concentration lead to the longest shoots, the highest leaf number, and high chlorophyll content (rules 13, 17, and 25, respectively; Table 5). As for Hard, only mineral solutions supplemented with a high concentration of Mg^2+^ (rules 9, 19, and 27; Table 5) promoted high values for SL, TLN, and LCC. According to these rules, the hardy kiwi plants grown on mineral formulation B containing high concentrations of NH_4_^+^, Cu^2+^, and Mg^2+^, corresponded to the longest SL (7.35 cm), the highest TLN (56.8 cm), and the second highest (almost nine CCI) LCC (Table 2).

Leaf area was essentially predicted by the interaction of NH_4_^+^ and Mg^2+^: if high NH_4_^+^ was combined with high Mg^2+^, then high LA values (rule 33; Table 5) were achieved, but if high NH_4_^+^ was combined with low Mg^2+^, then low LA values were obtained (rule 32; Table 5). Also, the model pinpointed the negative independent influence of NO_3_^–^, only promoting the highest LA when low amount of NO_3_^–^ was supplied into the mineral formulation (rule 34; Table 5; membership 1.00).

### Physiological Disorders

**FIGURE 2:**
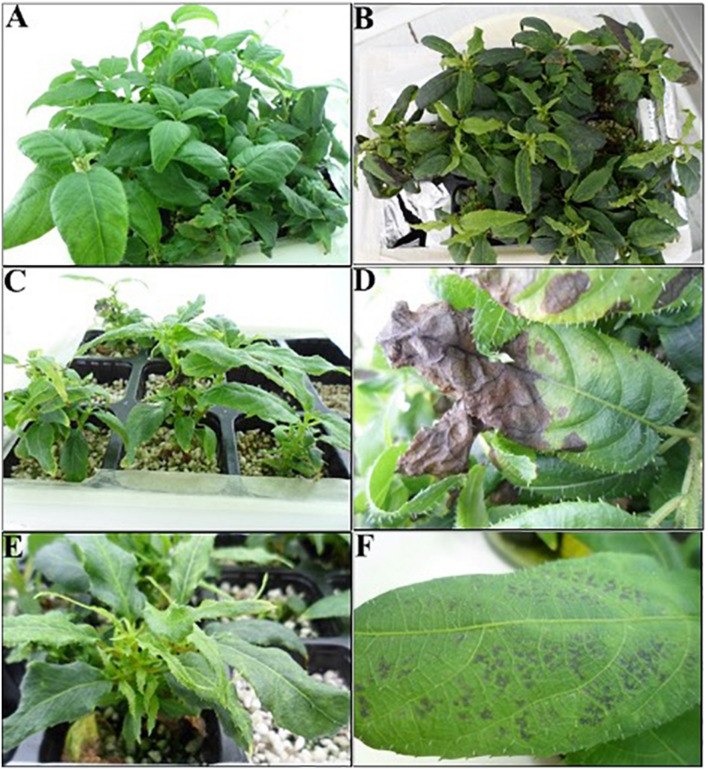
Growth quality of *A. arguta* plants watered with some mineral formulations in addition to physiological disorders observed in suboptimal media. **(A–C)** plants watered with **(A**,**B)**, and control formulations, respectively. Physiological disorders detected during plant acclimatization: **(D)** leaf necrosis, **(E)** curling leaf, and **(F)** leaf spot symptoms.

In this study, different types of physiological disorders such as LN, CL, and LS during plant acclimatization were observed (Table 3 and Figure 2). However, only LN could be successfully modeled with the neuro-fuzzy logic, due to insufficient predictability of their models (*f* ratio < *f* critical, Table 4).

Leaf necrosis variability is explained by Ca^2+^ concentration. The mid-high concentration of this ion (>1.03 mM) avoids leaf necrosis (rules 37–38; Table 5).

## Discussion

Although Hoagland solution (Hoagland and Arnon, 1950) has basically been set up using asparagus, lettuce, tomato, or wheat (Hoagland, 1923; Arnon, 1937; Arnon and Stout, 1939; Hoagland and Arnon, 1948), subsequently, it has been widely used to irrigate almost all genotypes, including several *Actinidia* spp. (Sotiropoulos et al., 2005; Liang et al., 2018; Liu et al., 2019; Purohit et al., 2020). In this study, we have used an IV-optimal design space using DOE software through dividing all macroelements plus copper of Hoagland solution into five independent factors at three levels (Table 1) to get a better understanding of the responses of acclimatized plants. This approach allowed us to (i) establish a well-sampled design space and (ii) reduce the number of mineral formulations based on Hoagland levels used in the study from 243 to just 19 combinations.

Among the available machine learning algorithms used in plant nutrition studies (García-Pérez et al., 2020; Hameg et al., 2020; Niazian and Niedbała, 2020), here, the commercial neuro-fuzzy logic (FormRules^®^) that combines artificial neural networks with fuzzy logic was used to build the mathematical models.

Neuro-fuzzy logic has shown a sounding potential for data mining and generates knowledge from complex datasets of plant tissue culture studies (Gago et al., 2011; Nezami-Alanagh et al., 2019). In this work, the efficiency of this tool can be briefly summarized as (i) generating statistical mathematical models with high predictability (train set *R*^2^ > 70%) and accuracy (*f* ratio > *f* critical), which explain six out of the eight *outputs* with the related significant *inputs* (Table 4) as described by Shao et al. (2006) and (ii) constructing a set of “IF–THEN” rules to elucidate the complex non-linear relationships between *inputs* and *outputs* expressed in words (Table 5). As an example, all the plants irrigated with C, H, J, and R formulations did not survive and died within the first 3 weeks of the acclimatization process mainly due to the low Ca^2+^ content (Table 3), all of them showing the highest (100%) leaf necrosis ratios (Table 5: rules 1 and 36).

Nitrogen (N) is considered one of the essential mineral nutrients for plant growth and development (Raven, 2003; Wang et al., 2012; Liu et al., 2014). Among the N sources, NH_4_^+^ and NO_3_^–^ ions are considered the most important, but plant species have different adaptabilities to uptake and utilize both (Crawford, 1995; Boudsocq et al., 2012; Gonçalves Fernandes, 2019; Asim et al., 2020). All the growth parameters studied were critically affected by one of the N sources or both, independently or in complex interactions with other ions such as Cu^2+^ or Mg^2+^ (Table 4). NO_3_^–^ should be supplied at a low concentration within the design space (6 mM) to improve both Hard and LA, whereas NH_4_^+^ should be supplied at high concentrations (0.5 < × < 1.0 mM) in combination with high Cu^2+^ or Mg^2+^ to improve SL, TLN, LCC, and LA (Table 5). Thus, the results also suggest that the concentrations of NH_4_^+^, Cu^2+^, and Mg^2+^ in the Hoagland solution are optimal to irrigate *Actinidia* spp., particularly kiwiberry (*A. arguta*). However, the concentrations of salts containing NO_3_^–^ should be reduced. In agreement with our findings, Clark et al. (2003) observed the preference of cranberry plants to absorb NH_4_^+^ compared with NO_3_^–^ when testing different ratios of NO_3_^–^:NH_4_^+^ based on Hoagland solution. In their study, the plants receiving NO_3_^–^ exhibited poorer growth and greater foliar chlorosis compared with plants grown with NH_4_^+^. In a recent study (Gonçalves Fernandes, 2019), in which *Actinidia* sp. plants were irrigated with two levels of these ions (0 and 3 mM) while keeping other mineral nutrients of solution at a fixed concentration, an increase in the lengths of shoots and roots was observed when solutions supplemented with 3 mM NO_3_^–^ were used, while the plants irrigated with 3 mM NH_4_^+^ exhibited higher chlorophyll and protein contents. These controversial results may have their origin in the experimental design, which in our case allows us to detect interactions between nitrogen suppliers and other ions, which undoubtedly influence the preference of plants in the absorption of certain nutrients.

Neuro-fuzzy logic also identified Mg^2+^ as an ion, which positively affected five out of the six parameters (Hard, SL, TLN, LCC, and LA) independently or in complex interaction with other ions (Table 4). Mg^2+^ was widely described as an essential divalent cation for plant growth and development, being considered as a mobile element (Tang et al., 2012; Blasco et al., 2015; Rehman et al., 2018; Hauer-Jákli and Tränkner, 2019). It is a structural constituent of chlorophyll molecules and the subsequent transport of photo-assimilates, and it is involved in many biochemical and physiological plant processes. It is also required for the activity of many enzymes of respiration and nucleic acid biosynthesis. The absence of magnesium results in interveinal chlorosis and in premature leaf abscission (Bhatla and Lal, 2018).

Recently, it has been shown that Mg^2+^ uptake by *Actinidia* sp. is significantly affected by N-sources available in the solutions, thus plants, whose source of N is exclusively NH_4_^+^, present lower Mg values in shoots and roots compared with plants irrigated with NO_3_^–^ (Gonçalves Fernandes, 2019). The antagonistic effect between Mg^2+^ and NH_4_^+^ may have its origin in uptake competition of these cations through the mechanism of charge balance in ion uptake, since N is a dominant macronutrient and its ionic form controls cation and anion uptake (Borgognone et al., 2013; Gonçalves Fernandes, 2019).

Ca^2+^ significantly affected three out of the six parameters. Despite the inclusion of a mid-high amount of this cation improving Hard and alleviating LN, the LCC was negatively influenced by high Ca^2+^ concentrations (Table 5). The dual function of calcium in plants, as the divalent cation, can be summarized in (i) contributing to the cell wall structure and strength and (ii) being a second messenger in many physiological and developmental processes (Thor, 2019). On the contrary, the visual symptoms of Ca^2+^ deficiency vary among species (de Freitas et al., 2016). In grapevine, it has been related to the appearance of necrosis at the margin of young leaves and the development of necrotic dots, rolling up, leaden, and yellow color in adult leaves (Bavaresco et al., 2010). We have observed leaf tip burning along with the development of necrosis in whole leaves when low amounts of Ca^2+^ were included in the formulations (Figure 2D). Recently, Teixeira da Silva et al. (2020) reviewed the effect of different ions on shoot tip necrosis (STN) in terms of morphological, biochemical, and molecular aspects, revealing that of all the ions, supply of sufficient Ca^2+^ *in vitro* cultures can prevent STN by inhibiting the accumulation of phenolic compounds and thus programmed cell death. Furthermore, the excessive synthesis and transport of auxin and ethylene in undesirable conditions were shown to decrease the mobility of Ca^2+^within a plant, resulting in Ca^2+^ deficiency and STN. They finally concluded that STN of *in vitro* shoots and/or plantlets can be hindered or reversed by altering the basal medium, mainly the concentration of Ca^2+^, adjusting the levels of auxins or cytokinins, or modifying culture conditions.

The copper ion (Cu^2+^) is considered an essential micronutrient that governs several important physiological roles during plant growth and development, mainly through its catalytic role in photosynthesis, respiration, and formation of lignin in the cell wall (Cuypers et al., 2000; Borghi et al., 2008; Printz et al., 2016). Our results pinpointed that this ion played an essential role in three out of the eight parameters (SL, TLN, and LCC) in interaction with NH_4_^+^. Both ions supplied at high concentration always (membership = 1) lead to long shoots, many leaves, and high chlorophyll contents. However, high Cu^2+^ concentrations combined with low NH_4_^+^ contents promote the opposite effect: short shoots, low leaf number, and chlorophyll content. These results suggest that the effect described on those growth and physiological parameters was more related to the level of NH_4_^+^ than the effect *per se* of the Cu^2+^.

In conclusion, the use of a reduced experimental design together with artificial intelligence tools has allowed us to study the simple or combined effect of nutrients in complex mineral formulations. Moreover, it has allowed us to establish the suitability of the full-strength Hoagland solution or propose its adjustment for better growth of the *A. arguta* plant during its acclimatization.

The nitrogen ions (NH_4_^+^ and NO_3_^–^) are essential to maintain plant growth and development. While the mathematical model obtained recommends maintaining the level of NH_4_^+^, Ca^2+^, Mg^2+^, and Cu^2+^ established in the full-strength Hoagland solution for irrigating kiwiberry plants, the NO_3_^–^ concentration should be reduced for improving plant hardening (e.g., at half the KNO_3_^–^ salt content of Hoagland solution).

## Data Availability Statement

The original contributions presented in the study are included in the article/Supplementary Material, further inquiries can be directed to the corresponding author/s.

## Author Contributions

SM performed the experiments. SM, BMZ, BK, and PG conceived and designed the experiments. SM, ML, and PG performed the DOE and machine learning models. SM and PG wrote the first draft of the manuscript. All authors contributed to manuscript revision, and read and approved the submitted version.

## Conflict of Interest

The authors declare that the research was conducted in the absence of any commercial or financial relationships that could be construed as a potential conflict of interest.

## Publisher’s Note

All claims expressed in this article are solely those of the authors and do not necessarily represent those of their affiliated organizations, or those of the publisher, the editors and the reviewers. Any product that may be evaluated in this article, or claim that may be made by its manufacturer, is not guaranteed or endorsed by the publisher.
