# Supplementary material for: Computer-Based Tools Unmask Critical Mineral Nutrient Interactions in Hoagland Solution for Healthy Kiwiberry Plant Acclimatization
Source: Front Plant Sci. 2021 Oct 28;12:723992. doi: 10.3389/fpls.2021.723992 (PMC8580943; doi:10.3389/fpls.2021.723992)
Supplement: Supplementary file 1 [file Data_Sheet_1.docx]

**SUPLEMENTARY TABLE 1**| The macro- and micro-elements combination in mg L^-1^ used for irrigation of *ex vitro* acclimatized plants.

| **Formulation** | **NH_4_H_2_PO_4_** | **Ca(NO_3_)_2_.4H_2_O** | **KNO_3_** | **MgSO_4_.7H_2_O** | **CuSO_4_.5H_2_O** | **H_3_BO_3_** | **ZnSO_4_.7H_2_O** | **Na_2_MoO_4_.2H_2_O** | **MnCl_2_.4H_2_O** | **FeSO4.7H_2_O** | **Na_2_EDTA.2H_2_O** |
| --- | --- | --- | --- | --- | --- | --- | --- | --- | --- | --- | --- |
| A | 57.515 | 944.6 | 6.066 | 492.9 | 0.0008 | 1.43 | 0.11 | 0.012 | 0.89 | 13.9 | 18.6 |
| B | 115.03 | 472.3 | 303.3 | 246.45 | 0.08 | 1.43 | 0.11 | 0.012 | 0.89 | 13.9 | 18.6 |
| C | 1.1503 | 9.446 | 606.6 | 4.929 | 0.04 | 1.43 | 0.11 | 0.012 | 0.89 | 13.9 | 18.6 |
| D | 1.1503 | 944.6 | 303.3 | 246.45 | 0.04 | 1.43 | 0.11 | 0.012 | 0.89 | 13.9 | 18.6 |
| E | 57.515 | 9.446 | 6.066 | 4.929 | 0.08 | 1.43 | 0.11 | 0.012 | 0.89 | 13.9 | 18.6 |
| F | 115.03 | 472.3 | 606.6 | 492.9 | 0.0008 | 1.43 | 0.11 | 0.012 | 0.89 | 13.9 | 18.6 |
| G | 57.515 | 944.6 | 303.3 | 492.9 | 0.0008 | 1.43 | 0.11 | 0.012 | 0.89 | 13.9 | 18.6 |
| H | 1.1503 | 9.446 | 606.6 | 246.45 | 0.08 | 1.43 | 0.11 | 0.012 | 0.89 | 13.9 | 18.6 |
| I | 115.03 | 472.3 | 6.066 | 4.929 | 0.04 | 1.43 | 0.11 | 0.012 | 0.89 | 13.9 | 18.6 |
| J | 1.1503 | 9.446 | 606.6 | 492.9 | 0.08 | 1.43 | 0.11 | 0.012 | 0.89 | 13.9 | 18.6 |
| K | 115.03 | 944.6 | 6.066 | 246.45 | 0.0008 | 1.43 | 0.11 | 0.012 | 0.89 | 13.9 | 18.6 |
| L | 57.515 | 472.3 | 303.3 | 4.929 | 0.04 | 1.43 | 0.11 | 0.012 | 0.89 | 13.9 | 18.6 |
| M | 57.515 | 944.6 | 6.066 | 492.9 | 0.08 | 1.43 | 0.11 | 0.012 | 0.89 | 13.9 | 18.6 |
| N | 1.1503 | 472.3 | 606.6 | 246.45 | 0.04 | 1.43 | 0.11 | 0.012 | 0.89 | 13.9 | 18.6 |
| O | 1.1503 | 472.3 | 303.3 | 492.9 | 0.08 | 1.43 | 0.11 | 0.012 | 0.89 | 13.9 | 18.6 |
| P | 115.03 | 944.6 | 606.6 | 246.45 | 0.04 | 1.43 | 0.11 | 0.012 | 0.89 | 13.9 | 18.6 |
| Q | 115.03 | 472.3 | 6.066 | 246.45 | 0.0008 | 1.43 | 0.11 | 0.012 | 0.89 | 13.9 | 18.6 |
| R | 1.1503 | 9.446 | 606.6 | 492.9 | 0.04 | 1.43 | 0.11 | 0.012 | 0.89 | 13.9 | 18.6 |
| S | 57.515 | 944.6 | 303.3 | 4.929 | 0.08 | 1.43 | 0.11 | 0.012 | 0.89 | 13.9 | 18.6 |
| Control | 57.515 | 472.3 | 303.3 | 246.45 | 0.04 | 1.43 | 0.11 | 0.012 | 0.89 | 13.9 | 18.6 |

**SUPLEMENTARY TABLE 2**| The macro- and micro-elements combination expressed as ion concentration (mM) used for irrigation of *ex vitro* acclimatized plants.

| **Formulation** | **NH_4_^+^** | **NO_3_^-^** | **K^+^** | **Ca^2+^** | **Mg^2+^** | **PO_4_^2-^** | **SO_4_^2-^** | **Cu^2+^** | **Mn^2+^** | **Zn^2+^** | **Na^+^** | **Fe^2+^** | **MoO_4_^2-^** | **BO_3_^-^** | **Cl^-^** | **EDTA^-^** |
| --- | --- | --- | --- | --- | --- | --- | --- | --- | --- | --- | --- | --- | --- | --- | --- | --- |
| A | 0.50 | 8.06 | 0.06 | 4.00 | 2.00 | 0.50 | 2.05 | 0.0000 | 0.0045 | 0.0004 | 0.0999 | 0.05 | 0,00005 | 0.023 | 0.009 | 0.05 |
| B | 1.00 | 7.00 | 3.00 | 2.00 | 1.00 | 1.00 | 1.05 | 0.0003 | 0.004497 |  |  |  |  |  |  |  |
| C | 0.01 | 6.08 | 6.00 | 0.04 | 0.02 | 0.01 | 0.07 | 0.0002 | 0.004497 |  |  |  |  |  |  |  |
| D | 0.01 | 11.00 | 3.00 | 4.00 | 1.00 | 0.01 | 1.05 | 0.0002 | 0.004497 |  |  |  |  |  |  |  |
| E | 0.50 | 0.14 | 0.06 | 0.04 | 0.02 | 0.50 | 0.07 | 0.0003 | 0.004497 |  |  |  |  |  |  |  |
| F | 1.00 | 10.00 | 6.00 | 2.00 | 2.00 | 1.00 | 2.05 | 0.0000 | 0.004497 |  |  |  |  |  |  |  |
| G | 0.50 | 11.00 | 3.00 | 4.00 | 2.00 | 0.50 | 2.05 | 0.0000 | 0.004497 |  |  |  |  |  |  |  |
| H | 0.01 | 6.08 | 6.00 | 0.04 | 1.00 | 0.01 | 1.05 | 0.0003 | 0.004497 |  |  |  |  |  |  |  |
| I | 1.00 | 4.06 | 0.06 | 2.00 | 0.02 | 1.00 | 0.07 | 0.0002 | 0.004497 |  |  |  |  |  |  |  |
| J | 0.01 | 6.08 | 6.00 | 0.04 | 2.00 | 0.01 | 2.05 | 0.0003 | 0.004497 |  |  |  |  |  |  |  |
| K | 1.00 | 8.06 | 0.06 | 4.00 | 1.00 | 1.00 | 1.05 | 0.0000 | 0.004497 |  |  |  |  |  |  |  |
| L | 0.50 | 7.00 | 3.00 | 2.00 | 0.02 | 0.50 | 0.07 | 0.0002 | 0.004497 |  |  |  |  |  |  |  |
| M | 0.50 | 8.06 | 0.06 | 4.00 | 2.00 | 0.50 | 2.05 | 0.0003 | 0.004497 |  |  |  |  |  |  |  |
| N | 0.01 | 10.00 | 6.00 | 2.00 | 1.00 | 0.01 | 1.05 | 0.0002 | 0.004497 |  |  |  |  |  |  |  |
| O | 0.01 | 7.00 | 3.00 | 2.00 | 2.00 | 0.01 | 2.05 | 0.0003 | 0.004497 |  |  |  |  |  |  |  |
| P | 1.00 | 14.00 | 6.00 | 4.00 | 1.00 | 1.00 | 1.05 | 0.0002 | 0.004497 |  |  |  |  |  |  |  |
| Q | 1.00 | 4.06 | 0.06 | 2.00 | 1.00 | 1.00 | 1.05 | 0.0000 | 0.004497 |  |  |  |  |  |  |  |
| R | 0.01 | 6.08 | 6.00 | 0.04 | 2.00 | 0.01 | 2.05 | 0.0002 | 0.004497 |  |  |  |  |  |  |  |
| S | 0.50 | 11.00 | 3.00 | 4.00 | 0.02 | 0.50 | 0.07 | 0.0003 | 0.004497 |  |  |  |  |  |  |  |
| Control | 0.50 | 7.00 | 3.00 | 2.00 | 1.00 | 0.50 | 1.05 | 0.0002 | 0.004497 |  |  |  |  |  |  |  |
